# Spectacularly Successful Microsurgical Penile Replantation in an Assaulted Patient: One Case Report

**DOI:** 10.1155/2011/865489

**Published:** 2011-11-16

**Authors:** Mohammed Fadl Tazi, Youness Ahallal, Abdelhak Khallouk, Mohammed Jamal Elfassi, Moulay Hassan Farih

**Affiliations:** ^1^Department of Urology, Hassan II Teaching Hospital, Fes 3000, Morocco; ^2^152, Lotissement Zouhour, VN, Meknes 47000, Morocco

## Abstract

Penile amputation is a rare condition for which immediate surgical replantation is warranted. We present herein one case of a 27-year-old male who presented to the Emergency Department after his wife cut his penis. The penis was replanted microsurgically. The deep dorsal penile veins and superficial veins were anastomosed. Although we could not reanastomose the arteries, wound healing occurred without any problem one week postoperatively and the patient regained erectile function 4 weeks after surgery. At 1-year follow-up examinations he reported on restored erectile function and a normal urinary function.

## 1. Introduction

Penile amputation is a rare urologic condition. It can occur as a result of self-mutilation of psychiatric patients, accidents, circumcision, and workplace injury, or it can be caused by other people's actions such as violence, envy, and crime [1, 2]. In 1970, in Thailand, an epidemic was seen, of penile amputation as punishment for philandering by humiliated wives [3]. Microvascular penile replantation offers the best prospect for restoration of urinary and erectile functions [4].

We present one case of spectacularly successful replantation of a penis without arterial anastomosis.

## 2. Case Presentation

A 27-year-old Moroccan male presented to the Emergency Department after being assaulted by his ex-wife. She cut off his penis using a shaving blade 1 cm distal from the mons pubis. She claimed she did so for revenge after she discovered his so-called infidelity even though they were already divorced. Immediately after the penile mutilation, the patient kept his amputated penis in a clean iced plastic. The patient presented at our institution 4 hours later. In initial evaluation, we found a clear cut through all penile structures without major lacerations. There were diffuse bleedings from the cavernosal bodies and an arterial and venous bleeding from the dorsal vessels.

**Figure 1:**
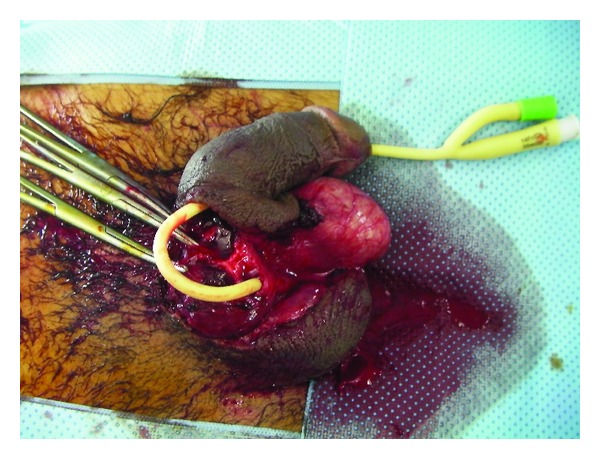
The amputated part is put on an 18F silicone Foley catheter which was passed into the patient's bladder.

The patient was, therefore, prepared for general anaesthesia; intravenous administration of 2 g Ceftriaxone along with 500 mg metronidazole was given. The patient had antitetanus serum and tetanus toxoid injection. As the patient had lost blood before his admission in Emergency Department, we transfused two unit each, of red blood cells (RBCs) and fresh frozen plasma (FFP). An immediate replantation of the amputated penis was attempted after a gross cleaning of the wound followed by meticulous debridement using an operating microscope with assistant optic: the amputated part was then put on an 18F silicone Foley catheter which was passed into the patient's bladder (Figure 1). We reanastomosed the urethra and the cavernosal bodies first. The urethra was repaired by spatulated end-to-end anastomosis with interrupted 4-0 vicryl sutures. Then, the deep dorsal penile vein was exposed, and a microsurgical end-to-end anastomosis was performed with 7-0 prolene sutures after irrigation with heparinized saline. The deep corporeal arteries and the dorsal penile arteries were identified but not anastomosed. The tunica albuginea of both corpora cavernosa and the septum were attached by running suture using 3-0 vicryl. Buck's fascia was closed with 4-0 vicryl, and then, the superficial vein was anastomosed with 7-0 prolene.

**Figure 2:**
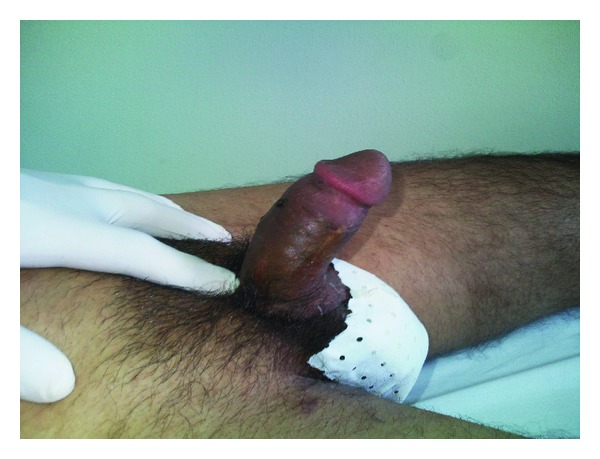
The patient regained his erectile function 4 weeks after the surgery.

Immediately the penile glans showed a sufficient perfusion. As a last step, the skin was readapted. A transurethral catheter was inserted for 21 days. Intravenous heparinized saline was given daily for one week to reduce blood viscosity and promote antithrombotic property. On the first postoperative week, wound healing occurred without any problem, no necrosis was noticed on the skin, and the patient regained his erectile function 4 weeks after the surgery (Figure 2). After one-year followup, the patient was fully satisfied with his body image and had a normal erectile and urinary function with good urine flow and absence of urethral stricture.

## 3. Discussion

Penile replantation was first described in the medical literature in 1929 [5]. The literature shows at least 30 cases of penile autoamputation with successful microsurgical replantation since 1970. In many of these cases, a restored erectile function and sensibility of the glans is stated within 1 y following the replantation [4, 5]. Because such a trauma is very rare, its management has evolved on the basis of only a few case reports and small series. Many factors contribute to favorable final outcomes: the degree of injury, type of injury (crushed, lacerated, or incised), duration of warm ischemia, the equipment used, and experience of the operative team [6]. Analysis of our case revealed that the cleanly incised injury with a short duration of cold ischemia was an important factor that influenced the outcome. Another factor was the concept of microsurgical replantation. The microsurgical approximation of the penile shaft structures provides early restoration of blood flow with the best prospects for graft survival, normal erectile function, and optimal benefits with fewer complications [3]. Our case study demonstrates, in the limit of a case report, that the venous drainage restoration for a good venous return is the most important factor in retrieving good postoperative results. In a series from Thailand, 14 of 18 replantations were done with a nonmicrosurgical technique. Skin loss was reported in 12 of 14 and graft loss in 6 of 14 patients [7]. Treatment of penile amputation has been greatly improved by microvascular techniques. Early restoration of blood flow, especially venous return as shown in our case, provides the best prospect for graft survival and normal erectile functional.

## 4. Conclusion

The current concept is that microsurgical reapproximation of the penile shaft structures provides the optimal benefit owing to having the fewest complications, the best prospects for cosmetic restoration, physiological micturition, and preservation of sensation and erectile function.
